# Retraction: Corrigendum: The association between diet and cardio-metabolic risk on cognitive performance: a cross-sectional study of middle-aged Australian adults

**DOI:** 10.3389/fnut.2024.1465534

**Published:** 2024-07-22

**Authors:** Frontiers Editorial Office

The journal retracts the February 28 2023 article cited above.

Following publication, the authors contacted the Editorial Office to request a second correction to their article due to errors in their coding. An investigation conducted in accordance with Frontiers' policies found that the findings reported in the article are no longer supported by the analysis. The article has been retracted and the authors are invited to present revised findings in a new manuscript.

The retraction of the article was approved by the Chief Editor of Frontiers in Nutrition and the Chief Executive Editor of Frontiers. The authors agree to this retraction.

